# GAS-GCN: Gated Action-Specific Graph Convolutional Networks for Skeleton-Based Action Recognition

**DOI:** 10.3390/s20123499

**Published:** 2020-06-21

**Authors:** Wensong Chan, Zhiqiang Tian, Yang Wu

**Affiliations:** 1 School of Software Engineering, Xi’an Jiaotong University, Xi’an 710049, China; cws201106004@stu.xjtu.edu.cn; 2 Division of Information Science, Nara Institute of Science and Technology, Ikoma 630-0192, Nara, Japan; yangwu@rsc.naist.jp

**Keywords:** deep learning, action recognition, graph convolutional networks, gated convolutional neural networks

## Abstract

Skeleton-based action recognition has achieved great advances with the development of graph convolutional networks (GCNs). Many existing GCNs-based models only use the fixed hand-crafted adjacency matrix to describe the connections between human body joints. This omits the important implicit connections between joints, which contain discriminative information for different actions. In this paper, we propose an action-specific graph convolutional module, which is able to extract the implicit connections and properly balance them for each action. In addition, to filter out the useless and redundant information in the temporal dimension, we propose a simple yet effective operation named gated temporal convolution. These two major novelties ensure the superiority of our proposed method, as demonstrated on three large-scale public datasets: NTU-RGB + D, Kinetics, and NTU-RGB + D 120, and also shown in the detailed ablation studies.

## 1. Introduction

With the wide use of the sensors like cameras and wearable devices, more and more information about our life is recorded. These sensory signals contain plenty of human action information. How to analyse these data to recognise human action has become a popular issue in recent years. Skeleton-based recognition is one of the research direction to solve this problem.

Skeleton-based action recognition has many applications in our life, such as human-computer interaction, intelligent monitoring, and industrial robots. Therefore, many methods for skeleton-based human action recognition are proposed in recent years [1,2,3,4,5,6,7]. Convolutional neural networks (CNNs) are abroad leveraged to extract the spatial-temporal features and generate classifications of action [8,9,10]. Recurrent neural networks (RNNs) are also used widely in skeleton-based action recognition because of their prominent ability to model long term dependencies in temporal dimension. Many RNN-based methods [2,11,12] achieve impressive results. Graph convolutional neural networks (GCNs) draw a lot of attention in recent years, because GCNs are expert in dealing with non-Euclidean data including skeleton data. Recent GCN-based methods [3,4,6,7,13] make great improvements in accuracy. These GCNs-based methods all adopt 1D convolutions in temporal dimension. Since RNNs are more powerful to handle temporal information than CNNs, some researches combine GCNs and RNNs together [5,14] for skeleton-based action recognition.

**Figure 1:**
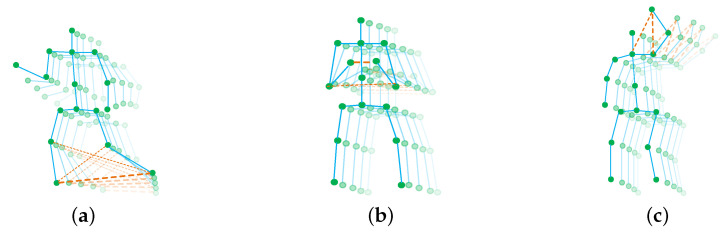
The illustration of action-specific edges. The green circles represent body joints. The blue solid lines represent the structural edges. The orange dashed lines represent the implicit edges. The thickness of lines represents the strength of relationship; (**a**) denotes the action-specific edges of the action running; (**b**) denotes the action-specific edges of the action clapping; (**c**) denotes the action-specific edges of the action waving hand. The action-specific edges are different for various actions.

As illustrated in Figure 1, the blue solid lines represent the structural edges. The orange dashed lines represent the implicit edges. Structural edges represent the natural connections of human body designed by handcraft. Implicit edges show the connections generated according to the input skeletons. Some joints are not connected with each other according to the structural edges. However, for some actions, these joints may be correlative. For instance, there should be strong connections between feet for the action running. The implicit edges will be able to describe these correlative connections. In addition, it is easy to neglect that the importances of implicit edges are different for various actions. For example, the implicit edges should be more necessary in rubbing two hands than in nodding head. Because the relationship between two hands is the key to recognize the rubbing. This relationship is represented by implicit edge. on the contrary, the motion between head and neck is the key to recognize nodding head. The connection between these two joints is structural edge. Therefore, the structural and implicit edges should be combined in different ratios for different actions. To solve this problem, we propose an action-specific graph convolutional module (ASGCM). The proposed ASGCM can generate the implicit edges and decide the ratio of the combination of structural edges and implicit edges according to different actions. The combinations of these two types of edges are action-specific edges. The action-specific edges will be used for graph convolution.

Skeleton-based action recognition also depends on the contexts and long-range dependencies in temporal dimension. Except for spatial information, the sequences of skeletons also contain many temporal information. However, not all information in temporal dimension is useful for action recognition. For instance, for the action clapping, the motion of hands in temporal dimension is important for action recognition, while the motion of other body parts are useless. Most of the previous researches do not pay enough attention to model temporal information in skeleton-based action recognition. For normal 1D temporal convolution, there might be many useless information in networks. Moreover, for normal 1D temporal convolution, the useful information might vanish in propagation. To control the information passing between different layers, a gated convolution network [15] is applied to model temporal information. Gating mechanism is usually used to control the information flowing in recurrent neural networks. Therefore, a gated CNN is presented in the proposed method to model the temporal information. To the best of our knowledge, this is the first work to adopt gated CNN in temporal dimension for action recognition.

Integrating these two major novelties, we propose a new model named gated action-specific graph convolutional network (GAS-GCN), in which ASGCM is applied to process the spatial information and the gated convolutional neural network operates in the time dimension. The experiments were performed on three large-scale datasets: NTU-RGB + D [16], NTU-RGB + D 120 [17], and Kinetics [18]. The main contributions of the proposed work can be summarized as follows:

1. We propose an action-specific graph convolutional module, which combines the structural and implicit edges together and also automatically learns the ratio of them according to the input skeleton data.
2. To filter out useless and redundant temporal information, we propose a gated CNN in the temporal dimension. The channel-wise attention is adopted in both GCN and gated CNN for information filtering.
3. A residual and cascaded framework is designed to integrate these two major components, which enhances and fuses their strengths. The proposed method outperforms state-of-the-art methods on NTU-RGB + D, NTU-RGB + D 120, and Kinetics dataset for skeleton-based action recognition.

## 2. Related Work

Skeleton-based action recognition draws wide attention in recent years. Before the widely use of deep learning, traditional human action recognition methods usually focus on the design of hand-crafted features. The HOJ3D [19] uses the histograms of 3D joint locations to represent the postures. Vemulapalli et al. [1] propose to use curves in Lie group to model human body’s action.

Action recognition based on deep learning makes great achievements by using CNN and RNN in recent years. Ke et al. [20] divide skeleton sequence to clips and use CNN to learn the spatial-temporal features. Song et al. [2] propose an end to end model based on LSTM and attention mechanism.

There are a lot of limitations for CNNs to deal with graph structure data. In recent years, graph convolutional networks are widely used in graph structure data since GCNs can outperform CNNs in non-Euclidean data [21,22,23,24,25]. It is natural to use graph convolutional networks in skeleton-based action recognition. Since the skeleton data can be easily seen as graph structure. ST-GCN [3] first applies graph convolution in spatial skeleton and 1D temporal convolution to deal with sequence information. Based on ST-GCN, PB-GCN [4] introduces a part-based graph convolutional network by separating skeleton architecture into serval body parts. However, in ST-GCN and PB-GCN, only structural edges exists in the graph networks. This may cause the lack of some vital information as we mentioned before. Therefore, 2s-AGCN [6] proposes the adaptive graph convolutional module to update the adjacency matrix according to the input of the networks. Furthermore, 2s-AGCN adopts two-stream structure to promote the recognition accuracy. Although 2s-AGCN combines the structural and implicit edges together, it ignores the ratio of these combinations. AGC-LSTM [5] combines graph convolution and LSTM together with attention mechanism. The gating mechanism in LSTM can control temporal information passing in propagation. However, compared with LSTM, the gated CNN used in this paper can reduce the gradient vanishing problem as mentioned by Dauphin et al. [15].

## 3. Methods

In this paper, we introduce a GCNs-based method for skeleton-based action recognition. In this section, a brief introduction about graph neural networks will be first given. Then, the proposed action-specific graph convolutional module will be represented in detail. Whereafter, we are going to show how the gated CNNs come into play in our method. In the end, the overall architecture and two-stream fusion of the proposed GAS-GCN will be shown.

### 3.1. Graph Convolutional Networks

In recent years, graph convolutional neural networks have played a very important role in processing graph structure data. Skeleton data can be naturally regarded as graph data. In skeleton data, the location of joints can be seen as the vertices in graph, and also the body connections of joints can be seen as edges. Skeleton data can be defined as graph G=(V,E). $V=\{v_{i},i=1,2,3,\ldots ,N\}$ denotes the set of *N* vertices. $E=\{e_{j},j=1,2,3,\ldots ,M\}$ denotes the set of *M* edges, in which $e_{j}$ is the connection of two vertices. Let the X denote the 2D or 3D coordinates of joints in skeleton data. The adjacency matrix A is used to aggregate the information of the neighboring nodes. The graph convolution operation f(X,A) in layer l+1 can be formulated as follows:

(1) $$H^{\left(l+1\right)}=\sigma \left(D^{-\frac{1}{2}}\tilde{A}D^{-\frac{1}{2}}H^{\left(l\right)}W^{\left(l\right)}\right)$$

where $\tilde{A}=A+I_{N}$ is the adjacency matrix of graph G with self-connections identity matrix $I_{N}$. $D_{ii}=\sum_{j}{\tilde{A}}_{ij}$ is the degree matrix. $H^{\left(l\right)}$ is the output feature of layer *l*, and $H^{0}=X$. $W^{\left(l\right)}$ is the trainable weight matrix. σ(·) denotes the activation function.

### 3.2. Action-Specific Graph Convolutional Module

Many existing graph convolutional networks only contain the structural edges designed by hand-crafted. This may cause some lose of implicit information. As we discussed in previous section, there might be some implicit edges between joints in action recognition. Moreover, the importance of implicit edges is different for various actions. Therefore, the ratio of the implicit and structural edges is also pretty vital. The proposed action-specific graph convolution module can generate the implicit edges and the ratio of the implicit and structural edges according to the input feature.

**Figure 2:**
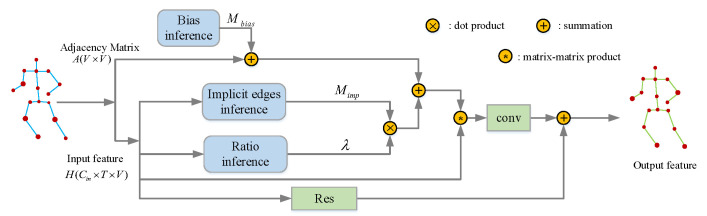
The illustration of the action-specific Graph Convolution Module. H is the input feature with the size of $C_{in}\times T\times V$. $A\in R^{V\times V}$ is the adjacency matrix. The implicit edges inference will produce the implicit connections $M_{imp}$. The ratio inference is also implemented by Equation (4). It will learn a weight λ to decide the ratio of $M_{imp}$. $M_{bias}$ is the learnable matrix as the bias, which is produced by bias inference. These three matrixes will be added together as the adjacency matrix of graph convolutional layer. The matrix–matrix product followed by conv denotes the graph convolution operation. The output dimension of this module will not be changed in spatial dimension and temporal dimension.

As illustrated in Figure 2, $H\in R^{C_{in}\times T\times V}$ is the input feature. Here $C_{in}$ is the number of channels. *V* is the number of nodes and *T* is the length of the skeleton sequence. $A\in R^{V\times V}$ is the adjacency matrix designed by handcraft, which can be regarded as the structural edges. Since handcrafted matrix A is insufficient to describe all structural edges, $M_{bias}^{\left(l\right)}$ is introduced as a supplement to A. Therefore, *A* and $M_{bias}^{\left(l\right)}$ present the structural edges. In particular, $M_{bias}$ is initialized with A and updated in back propagation. In our work, structural edges are same for different actions. The hyper-parameter μ restricts the adjustment range of the supplement. The adjustment range is determinate and same for different actions. Therefore, μ is manually set. The implicit edges inference will produce the implicit connections $M_{imp}\in R^{V\times V}$. The ratio inference will produce a scalar λ to decide the ratio between structural and implicit edges. The final adjacency matrix used to aggregate the information of neighbors in the proposed method can be formulated as follows in layer *l*:

(2) $$M_{imp}^{\left(l\right)}=softmax\left({H^{\left(l-1\right)}}^{T}W_{\theta }^{T}W_{\phi }H^{\left(l-1\right)}\right)$$

(3) $$M^{\left(l\right)}=A+\mu M_{bias}^{\left(l\right)}+\lambda^{\left(l\right)}M_{imp}^{\left(l\right)}$$

(4) $$\lambda^{\left(l\right)}=\frac{1}{VT^{l}}\sum_{i=1}^{T^{l}}\sum_{j=1}^{V}{\left(H^{\left(l-1\right)}W^{\left(l\right)}\right)}_{i,j}$$

where $M^{\left(l\right)}$ is the final adjacency matrix used in graph convolution in layer *l*. $H^{\left(l-1\right)}$ is the output feature of layer l−1, and $H^{\left(0\right)}=X$. $W_{\theta }$ and $W_{\phi }$ denote the 1×1 convolution operation with *C* convolution kernels. The outputs of these two convolutional operations have the same shape C×T×V. The outputs of these two convolution will be reshaped and multiplied with each other to generate $M_{imp}\in R^{V\times V}$. $W^{\left(l\right)}$ denotes the 1×1 convolution operation with only one convolution kernel. The output size of this convolution operation is $V\times T^{\prime }$. $T^{\prime }$ is the new size of the temporal dimension.

The action-specific adjacency matrix $M^{\left(l\right)}$ and the feature $H^{\left(l-1\right)}$ will be the inputs of the graph convolution operation formulated as Equation (1). The matrix–matrix product followed by conv in Figure 2 represents the graph convolution operation. The residual block is also adopted in this module to alleviate the gradient vanishing problem.

### 3.3. Gated Convolutional Neural Networks

**Figure 3:**
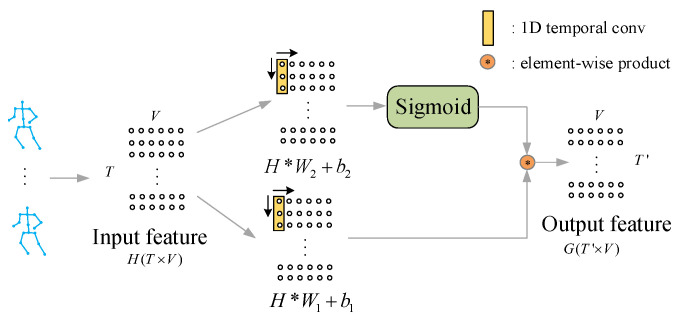
The illustration of gated convolution operation. The feature of skeleton sequences will be normalized as the matrixes H(T×V). These two 1D temporal convolutions (TC) have the same kernel size *k*. TC followed by the sigmoid function computes the value of gate mechanism. The output feature of another TC is multiplied by gates.

Besides modeling the spatial skeleton information in space dimension, it is also important to model the temporal information in time dimension. Gated convolutional networks is firstly proposed for natural language processing by Dauphin et al. [15]. The gate mechanism can be usually found in recurrent neural networks like LSTM. The gated CNN takes example by gate mechanism in LSTM. Different from LSTM, the gated CNN is more efficient in computations. The experiments results shown by Dauphin et al. [15] denote that the gated CNN achieves better performance than LSTM. In this work, the gated CNN is adopted to process the skeleton sequences. The architecture of gated CNNs is shown in Figure 3. These two 1D temporal convolutions (TC) have the same kernel size *k* and channel size *C*. The TC followed by sigmoid function is used to produce gates according to the context information in temporal dimension. The output feature of another TC multiplied by the gates will be the output feature of the gated convolution. The gates control the information passing between different layers. It is able to keep the useful information relevant to the recognition and abandon the useless information. The gated convolution operation used in the proposed method can be formulated as follows:

(5) $$G=\left(H*W_{1}+b_{1}\right)⊗sigmoid\left(H*W_{2}+b_{2}\right)$$

where $H\in R^{T\times V\times C_{in}}$ is the input feature from previous layer. $W_{1}$ and $W_{2}$ both denote the convolution kernels in temporal dimension with the same size. $b_{1}$ and $b_{2}$ are both the bias, which is trainable. The sigmoid function is the activation function used to generate gates. The operation ⊗ denotes the element-wise product. The output feature of TC multiplied by the gates is the final output feature G of the gated convolution.

### 3.4. The Gated Action-Specific Convolutional Networks

**Figure 4:**
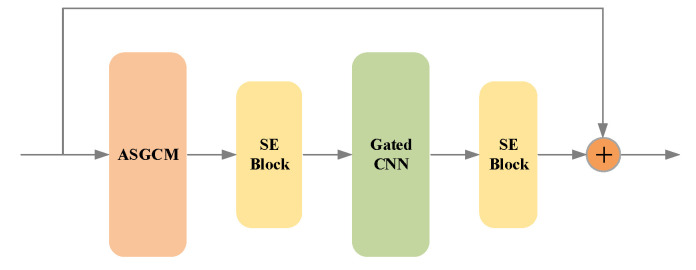
The gated action-specific convolution network. The ASGCM is used for spatial skeleton. The gated CNN is used for temporal sequences. The SE block is the channel-wise attention.

The action-specific graph convolution module and gated convolution will be combined in the proposed method. The block of the action-specific convolution layer in our proposed method is illustrated in Figure 4. The ASGCM is used to aggregate the spatial information in each frame. The gated CNN is 1D temporal convolution as we have discussed. The gated CNN is used to model the temporal information in skeleton sequences. SE block [26] is a channel wise attention mechanism. Since we assume that the features in different channels have various importance. The SE block is applied after ASGCM in our method. The SE block is also adopted after this gated convolution as the channel-wise attention. The residual block is also adopted to avoid the vanishing gradient.

### 3.5. The Architecture of the Networks

**Figure 5:**
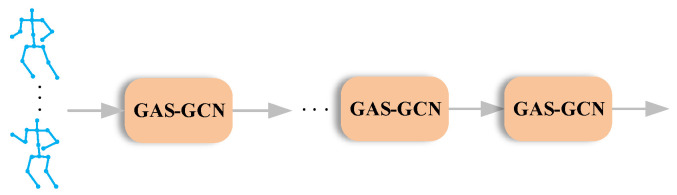
The architecture of the proposed method. The cascaded GAS-GCN is adopted in the proposed method. The input of the networks are skeleton data. At the end of the networks, FC layer is utilized to produce the classification score.

The cascaded GAS-GCN is proposed in our method. The overall architecture is shown in Figure 5. In our method, multi-GCNs have 10 layers. The first four layers have 64 output channels. The fifth to seventh layers have 128 output channel. The last three layers have 256 output channels. Only the strides of the fifth layer and the eighth layers are two while the rest layers are one in temporal dimension. Following the last layer, the FC layer is adopted to generate the final classification score.

**Figure 6:**
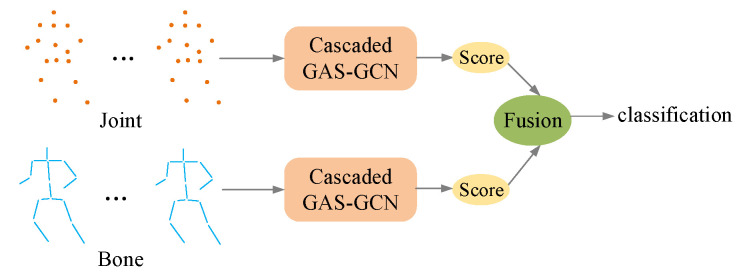
The illustration of two-stream fusion architecture. The two streams use different input data. They have the same architecture. Their prediction scores are added together as the final classification score.

Two-stream architecture can be seen in many convolutional networks. In our work, like 2s-AGCNs [6], the two-stream fusion method is also adopted to further boost the classification performance. Except for the first-order information, joint information, another stream of the networks is bone information. Bone information is the second-order information, which is also useful for the action classification. Joint information and bone information are used for the input of the two-stream network. One stream uses the joint information while another uses bone information. The two-stream architecture is shown in Figure 6. They have the same architecture illustrated in Figure 5. They are trained independently. In the test process, both streams produce the prediction scores. Then the scores from these two streams of each action sequence are added together to generate the final score. These two streams are complementary and can achieve better results in our experiments.

## 4. Experiments

To show the effectiveness of the proposed method, the experiments were performed on three large scale datasets: NTU-RGB + D [16], NTU-RGB + D 120 [17], and Kinetics [18]. In the beginning of this section, these three datasets are introduced briefly. Next, we are going to show the train details of our experiments. Then, our method will be compared with several state of the arts on NTU-RGB + D and Kinetics dataset. The results of the ablation experiments and some visualized results will be shown to analyze the proposed method in detail. At the end of this section, the accuracy of the proposed method on NTU-RGB + D 120 dataset will be reported. The code will be available on Github after the paper’s reviewing.

### 4.1. Datasets

**NTU-RGB + D** [16]. NTU-RGB + D contains 56,880 videos in total. This dataset consists of various modalities including RGB+D videos, IR videos and 3D joints. Since we focus on the skeleton-based action recognition, only the 3D joints data of NTU-RGB + D is used in the experiments. To provide a standard evaluation of all reported results on this benchmark, two types of evaluation metrics for this dataset are recommended, which are cross-view (CV) and cross-subject (CS). In cross-subject evaluation, the 40 subjects are split into training and testing groups. Each group consists of 20 subjects. The videos are captured by three cameras from different views. For cross-view evaluation, the samples of camera 1 are used for testing, while samples of cameras 2 and 3 are used for training.

**NTU-RGB + D 120** [17]. NTU-RGB + D 120 is an extension of NTU-RGB + D. The total number of videos in this dataset is 114,480. This dataset has 120 action classes. The evaluation metrics for this dataset are cross-subject and cross-setup.

**Kinetics** [18]. Kinetics is a much larger dataset than above two datasets. In this dataset, there are 306,245 videos in total. However, original Kinetics dataset only contains RGB videos without 3D joints information. Therefore, the extracted Kinetics-skeleton data by Yan et al. [3] is used for evaluation in our experiments. The evaluation metrics for this dataset are top-1 and top-5 accuracy. Top-1 accuracy means that the prediction (the one with the highest probability) of the model must be exactly the true label. Top-5 accuracy means that one of the five highest probability predictions of the model must match the true label.

### 4.2. Experiments Details

In our experiments, the proposed method is implemented by PyTorch [27]. To train and test conveniently, the fixed length *T* of skeleton sequence is sampled as input. The stochastic gradient descent (SGD) is applied in the training process as the optimization strategy. The code is run on two NVIDIA 2080TI GPUs with the memory of 11GB. Compared with AGCN, the cost of the GPU memory only increases about 20%. The consumption of time also increases about 20%. For NTU-RGB + D dataset, *T* is set as 300. The input of the networks are the coordinates of joints. Besides, temporal displacements and relative coordinates are also utilized as the complementary inputs [4] for the coordinates. The total training epoch is 50. The base learning rate is set as 0.1 at the first 30 epochs. Then, the learning rate will be multiplied by 0.1 in epoch 30 and epoch 40. The training batch size is 28. For Kinetics, *T* is set as 150. The training epoch is larger than NTU-RGB + D, which is 65. The base learning rate is also set as 0.1. Then, in epoch 45 and 55, the learning rate will be multiplied by 0.1. The training batch size is set as 64. μ is set as 0.5 in Equation (3).

### 4.3. Comparisons with the State of the Arts

To show the remarkable performance of the proposed method, the proposed gated action-specific graph convolution network (GAS-GCN) is compared with the state-of-the-art methods on NTU-RGB + D dataset and Kinetics dataset.

#### 4.3.1. NTU-RGB + D Dataset

The proposed method is firstly compared with some state-of-the-art methods on NTU-RGB + D dataset. The comparison results are shown in Table 1. The proposed method GAS-GCN achieves the accuracy of 90.4% in cross-subject evaluation and 96.5% in cross-view evaluation. The results of hand-craft methods, RNN-based methods, CNN-based methods, and GCN-based methods are listed in the table. It is obvious to see that the proposed method outperforms all these listed methods. Compared with the baseline method 2s-AGCN, the proposed method achieves 1.9% and 1.4% improvement in accuracy. It is worth to mention that DGNN [7] uses both 3D skeleton information and RGB information in their experiments. However, the proposed method is still superior to DGNN in terms of two evaluation metrics. Compared with state-of-the-art method AGC-LSTM [5], the proposed method also achieves better accuracy. This shows the effectiveness of the gated convolution operation in modeling temporal information for skeleton data.

#### 4.3.2. Kinetics Dataset

The proposed method is also compared with some advanced methods on Kinetics dataset. As shown in Table 2, the top-1 accuracy of the proposed method is 37.8% and top-5 is 60.9%. Hand-crafted methods, LSTM-based methods, and GCN-based methods are listed in the table. Compared with these state-of-the-art methods, the proposed method also achieves the best performance for both top-1 and top-5.

### 4.4. Ablation Experiments

#### 4.4.1. Ablation Results

An ablation experiment was performed to show the influence of ASGCM, gated CNN, and their assembly on NTU-RGB + D dataset.

The ablation results are shown in Table 3. Plain GAS-GCN achieves the accuracy of 94.34%. This is the baseline of the proposed method. Then, the experiment of the plain GAS-GCN with ASGCM is performed. The accuracy is 94.59%. The experiment of the plain GAS-GCN with gated convolution is also performed. The accuracy is 94.75%. These results demonstrates the effectiveness of the proposed ASGCM and the gated convolution. The proposed method with the assembly of ASGCM and gated convolution achieves the accuracy of 95.14%. The result shows that the combination of ASGCM and gated convolution can achieve the best performance. In addition, the two-stream results of AGCN and GAS-GCN are shown at the end of the table. It is worth to note that even the single stream of the GAS-GCN can achieve the better result than two-stream method AGCN.

**Figure 7:**
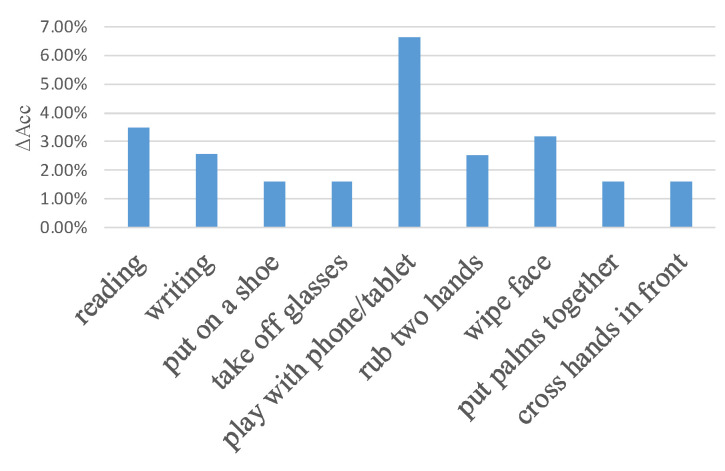
The accuracy promotion for several actions with ASGCM. ΔAcc denotes the promotion of accuracy.

To further show the effectiveness of the ASGCM, the accuracy promotion for several actions with ASGCM are shown in Figure 7. For instance, for actions rubbing two hands, putting palms together, and crossing hands in front, there should be implicit relationship between two hands as we mentioned before. Compared with the adaptive graph convolution of baseline [6], the proposed ASGCM can better model the implicit relationship between joints.

#### 4.4.2. The Results of Two-Stream Fusion

In this work, the two-stream fusion architecture is adopted to boost the performance of the proposed method. The two-stream fusion results are shown in Table 4 and Table 5. As we can see in Table 4, on NTU-RGB + D dataset, for the cross-subject evaluation, the accuracy of the proposed method is 95.1% with joint information and 95.0% with bone information. The fusion of these two streams achieves the accuracy of 96.5%. For the cross-view evaluation, the proposed method achieves the accuracy of 87.9% with joint information and 88.2% with bone information. The result of two-stream fusion for cross-view evaluation is 90.4%. These results of two-stream fusion on NTU-RGB + D dataset are both remarkable.

In addition, the two-stream fusion experiment on Kinetics dataset is also performed as illustrated in Table 5. The accuracy of joint information on Kinetics dataset is 36.1%. The accuracy of bone information is 35.6%. The two-stream fusion on Kinetics dataset achieves 37.8%. The results on NTU-RGB + D and Kinetics dataset show that the proposed method can achieve state-of-the-art results even only using joint or bone information. Based on this, the two-stream fusion can further boost the performance of the proposed method.

#### 4.4.3. Visualization of Action-Specific Graph Edges

**Figure 8:**
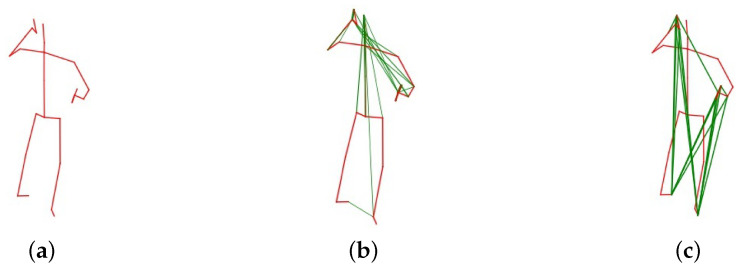
The visualization of the structural and action-specific edges on skeleton data. The thickness of the lines depicts the strength of relationship between two joints. The red lines denote the structural edges according to the body connection. The green lines represent the implicit edges generated by the proposed action-specific graph convolutional module; (**a**) illustrates the structural skeleton for action phone call; (**b**,**c**) illustrates the action-specific skeleton in layer 1 and 10 for action phone call. These two figures demonstrate the edges in different layers are various.

The visualization of the action-specific edges is illustrated in Figure 8. The thickness of the lines depicts the strength of relationship between two joints. The red lines denote the structural edges according to the body connection. The green lines represent the implicit edges generated by the proposed action-specific graph convolutional module. Most of the red lines are thicker that green lines. This means that although the ASGCM generates the implicit edges but the structural edges are still more important. The implicit edges are complementary to the structural edges. The comparison between Figure 8b,c demonstrates that the edges in different layers are various.

### 4.5. Results on NTU-RGB + D 120 Dataset

As far as we know, there are no other published results of recent methods on this dataset due to that the dataset has just been released. Therefore, we will not compare the proposed method with state-of-the-art methods. The results of the proposed method on NTU-RGB + D 120 are shown in Table 6. For cross-subject evaluation, the accuracy is 83.0% with joint information and 83.4% with bone information. The two-stream fusion method achieves the accuracy of 86.4%. For cross-setup evaluation, the proposed GAS-GCN achieves the accuracy of 85.0% with joint information and also 85.0% with bone information. The fusion of these two streams achieves the accuracy of 88.0%.

## 5. Conclusions

In this paper, we propose a gated action-specific graph convolutional network for skeleton-based action recognition. To capture the implicit edges in skeleton data, we propose an action-specific convolutional module. This module can combine the structural with implicit edges and also decide the ratio of these two types of edges. To model temporal information, a gated convolution operation is adopted to process the skeleton sequences in temporal dimension. The SE block and two-stream fusion architecture are also used in the proposed method to boost the performance. The proposed method achieves remarkable accuracy compared with several state-of-the-art methods on NTU-RGB + D, and Kinetics dataset. The alation results of the proposed method are presented to show the effectiveness of ASGCM and gated CNN. Futhermore, the results of the proposed method on NTU-RGB + D 120 dataset are also shown. The experiment results demonstrate the superiority of the proposed GAS-GCN. It is worth to mention that the action-specific graph convolution and gated convolution can be applied in many other computer vision tasks.

## Figures and Tables

**Table 1:** Comparison with the state-of-the-art methods on NTU-RGB + D dataset.

Methods	Year	Accuracy(%)	
		Cross-Subject	Cross-View
Lie Group [1]	2014	50.1	52.8
H-RNN [28]	2015	59.1	64.0
Part-aware LSTM [16]	2016	62.9	70.3
ST-LSTM [29]	2016	69.2	77.7
Two-stream RNN [30]	2017	71.3	79.5
STA-LSTM [2]	2017	73.4	81.2
Ensemble TS-LSTM [31]	2017	74.6	81.3
Visualization CNN [8]	2017	76.0	82.6
C-CNN + MTLN [20]	2017	79.6	84.8
Temporal Conv [9]	2017	74.3	83.1
VA-LSTM [32]	2017	79.4	87.6
Beyond Joints [12]	2018	79.5	87.6
ST-GCN [3]	2018	81.5	88.3
DPRL [33]	2018	83.5	89.8
HCN [34]	2018	86.5	91.1
SR-TSL [14]	2018	84.8	92.4
MAN [35]	2018	82.7	93.2
PB-GCN [4]	2018	87.5	93.2
RA-GCN [36]	2019	85.9	93.5
AS-GCN [13]	2019	86.8	94.2
AGC-LSTM [5]	2019	89.2	95.0
2s-AGCN [6]	2019	88.5	95.1
DGNN [7]	2019	89.9	96.1
GAS-GCN(Ours)	2020	90.4	96.5

**Table 2:** Comparison with the state-of-the-art methods on Kinetics dataset. The top-1 and top-5 accuracy of the proposed method and other state-of-the-art methods are shown.

Methods	Year	Accuracy(%)	
		Top-1	Top-5
Feature Enc [37]	2015	14.9	25.8
Deep LSTM [16]	2016	16.4	35.3
Temporal Conv [9]	2017	20.3	40.0
ST-GCN [3]	2018	30.7	52.8
AS-GCN [13]	2019	34.8	56.5
2s-AGCN [6]	2019	36.1	58.7
DGNN [7]	2019	36.9	59.6
GAS-GCN (Ours)	2020	37.8	60.7

**Table 3:** The importance of ASGCM (AG) and gated CNN (GN) were evaluated on NTU-RGB + D dataset. Plain GAS-GCN (PG) denotes the baseline of our method, i.e., without AG and GN.

Stream	Model	Accuracy (%)
Joint stream only	AGCN	93.83
	PG	94.34
	PG + AG	94.59
	PG + GN	94.75
	PG + AG + GN	95.14
Two stream	AGCN	95.10
	GAS-GCN	96.50

**Table 4:** Two-stream fusion results on NTU-RGB + D dataset.

Methods	CS (%)	CV (%)
GAS-GCN (Joint)	87.9	95.1
GAS-GCN (Bone)	88.2	95.0
GAS-GCN (Joint&Bone)	90.4	96.5

**Table 5:** Two-stream fusion results of Top-1 accuracy on Kinetics dataset.

Methods	Accuracy (%)
GAS-GCN (Joint)	36.1
GAS-GCN (Bone)	35.6
GAS-GCN (Joint&Bone)	37.8

**Table 6:** The experimental results on NTU-RGB + D 120 dataset. As far as we know, there are no other published results of recent methods on this dataset due to that the dataset has just been released.

Methods	Cross-Subject (%)	Cross-Setup (%)
GAS-GCN (Joint)	83.0	85.0
GAS-GCN (Bone)	83.4	85.0
GAS-GCN (Joint&Bone)	86.4	88.0
